# A comparative survey of Nigerian physiotherapists’ familiarity with, knowledge of and utilisation of standard outcome measures: 10 years after initial survey

**DOI:** 10.4102/sajp.v74i1.435

**Published:** 2018-06-28

**Authors:** Adesola C. Odole, Olufemi O. Oyewole, Aderonke O. Akinpelu

**Affiliations:** 1 Department of Physiotherapy, College of Medicine, University of Ibadan, Nigeria; 2 Department of Physiotherapy, Olabisi Onabanjo University Teaching Hospital, Nigeria

## Abstract

**Background:**

The need for physiotherapists to use standardised outcome measures (SOMs) is recognised and recommended in clinical practice guidelines in many countries.

**Aim:**

To evaluate changes in physiotherapy practice in Nigeria on the utilisation of SOMs and physiotherapists’ familiarity with and knowledge of SOMs over the past decade.

**Methods:**

A comparative cross-sectional survey of present data with 2006 data was undertaken. The existing validated questionnaire of 2006 was used to assess physiotherapists’ familiarity with, knowledge of and utilisation of 16 SOMs.

**Results:**

There was a noticeable change in familiarity with and utilisation of 16 SOMs in the current data and in knowledge. Between 52% and 90% of physiotherapists were not familiar with 14 SOMs in 2006, whereas 51.4% – 85.8% of physiotherapists were not familiar with 8 SOMs in 2016; 77% – 97% and 63.4% – 97.3% of physiotherapists were not utilising SOMs in the 2006 and 2016 data, respectively. The least utilised SOMs in 2006 were Western Ontario McMaster Osteoarthritis Index, Chedoke McMaster Stroke Assessment and SF-36 Health Survey; in 2016, it was only the Chedoke McMaster Stroke Assessment. The Visual Analogue Scale and Gross Motor Function Measure remained the most utilised in both data. Duration of practice, age and sex were significant factors for the utilisation of and familiarity with SOMs.

**Conclusion:**

There was an improvement in the familiarity with, knowledge of and utilisation of SOMs over the past decade among Nigerian physiotherapists but the level of utilisation is unsatisfactory. Action is required if routine outcome measurement is to be achieved.

**Clinical Implications:**

Utilisation of SOMs is part of core standards of physiotherapy practice for effective management of patients. Although the utilisation of SOMs improved over the past 10 years, it is very low. Therefore, studies directed at finding factors responsible for low utilisation of SOMs among Nigerian physiotherapists are warranted.

## Introduction

Measurement is a fundamental component of evidence-based medicine and provides the necessary information clinicians require to make decisions in patient management (Antunes, Harding & Higginson 2014). Thus, standardised outcome measures (SOMs) are used like a conventional laboratory test, that is, to inform the differential diagnosis and monitor a patient’s progress (Antunes et al. 2014). Outcome measures are assessments that measure change in patients’ functioning, performance or participation over time following an intervention (Copeland, Taylor & Dean 2008; Duncan & Murray 2012; MacDermid et al. 2013). Monitoring the health status of patients through the use of outcome measures is considered to be an aspect of good clinical practice in physiotherapy (Stevens & Beurskens 2010). Data generated through SOMs can be helpful in several phases of a condition or disease management: during the initial history and physical examination, laboratory and imaging procedures of investigation leading to a diagnosis, all phases of treatment and follow-up (Antunes et al. 2014).

In a review by Duncan and Murray (2012), several reasons for promoting SOMs have been stated. These include the following: (1) Routine outcome measurement provides clinicians with feedback on the types of achieved outcomes and how these outcomes compare with those of other health professionals; (2) Records of patient outcomes also enable progress, which can sometimes appear intangible, to be effectively communicated to patients and also promote efficient treatment planning; (3) Routine outcome measurement can also be used to support the clinical justification of interventions and provide important supporting evidence to health care funding bodies. This was buttressed in another study which reported that valid and reliable clinical outcome measurement can support better clinical decision-making, quality assurance or clinical research (MacDermid et al. 2013). Outcome measures alert clinicians about the severity of a symptom or disability and so contribute to their focus of treatment intervention (MacDermid et al. 2013). In another review, the authors reported that in palliative care settings, systematic collection of patient-reported outcome measures (PROM) using validated questionnaires may benefit clinical practice both at a population and individual patient level (Antunes et al. 2014). They postulated that this may be achieved by the following mechanisms: (1) facilitating identification and screening of physical, psychological, spiritual and social unmet needs that might otherwise be overlooked; (2) providing information on disease progression and impact of treatment prescribed; (3) facilitating patient–clinician or family–clinician or carer–clinician communication, promoting the model of patient-centred care by shared decision-making and advanced care planning, establishing common priorities and expectations regarding outcomes of treatment and disease progression, which does not mean using a PROM to act as a substitute for the therapeutic relationship, but rather to complement it; and (4) monitoring outcomes by performing audits as a strategy for improvement of the quality of care provided and its costs (Antunes et al. 2014).

The need for physiotherapists to use SOMs has been recognised at the national level in the United States (Jette et al. 2009). This has been articulated in a number of policy statements including the core standards of physiotherapy practice of the World Confederation of Physical Therapy (WCPT 2011). Adherence to this policy by Nigerian physiotherapists (i.e. utilisation of SOMs) was investigated in 2006 by evaluating the familiarity with, knowledge of and utilisation of SOMs (Akinpelu & Eluchie 2006). The authors suggested that physiotherapists in Nigeria have poor knowledge of, seldom utilise, and were unfamiliar with SOMs. They recommended that promotion of SOMs use among Nigerian physiotherapists could be enhanced through organising workshops on outcomes assessment for members by the Nigeria Society of Physiotherapy (NSP) and through inclusion of outcomes assessment into undergraduate physiotherapy curricula. They also recommended that physiotherapy researchers in Nigeria should validate existing outcome measures in their environment and develop new outcome measures that are Nigeria culture and environment friendly. Over the past decade, it was assumed that these recommendations should have been appreciably implemented. It appears though that there has not been any published study since that of 2006 investigating the state of Nigerian physiotherapists on the utilisation of SOMs. The primary purpose of this survey was to consider changes in physiotherapy practice in Nigeria on the utilisation of SOMs by physiotherapists working in various settings of rehabilitation as well as their familiarity with, general knowledge and knowledge of administration of SOMs over the past decade (2006–2016).

## Methods

This study was a cross-sectional survey aimed at comparing the present data with the data published in 2006. A representative sample of participants for this study was conveniently sampled: physiotherapists practising in various facilities (primary health care centres, state government–owned hospitals and university teaching hospitals, private physiotherapy clinics, homes and schools for children and adults with special needs, sports centres and physiotherapy training institutions) in Nigeria. The study used the same validated questionnaire as in 2006 (Akinpelu & Eluchie 2006). There are three parts to the questionnaire: the first part obtained demographic data of the participants, whereas the second part assessed familiarity with and utilisation of 16 SOMs. The third part assessed the knowledge of administration and general knowledge. The responses on familiarity with and utilisation of 16 SOMs were on 5-point Likert ordinal scales. In order to compare data from the two studies, responses of the participants on familiarity with and utilisation of SOMs were dichotomised into two groups: ‘Not familiar’ and ‘Barely familiar’ as ‘Not familiar’; ‘Familiar’, ‘Quite familiar’ and ‘Very familiar’ as ‘Familiar’”; ‘Never’ and ‘Rarely’ as ‘Not utilised’; and ‘Sometimes’, ‘Often’ and ‘Always’ as ‘Utilised’. The correct answer to method of administration was allotted one mark giving rise to a possible total of 16 points. The 16 SOMs selected in this study were different from the SOMs tested in previous published studies. It is assumed based on the burden of disability listed by the Institute for Health Metrics and Evaluation in Nigeria (http://www.healthdata.org/nigeria) that these SOMs are relevant to Nigeria and seem to support the expectation that practising physiotherapists in Nigeria should be aware of the 16 SOMs.

The 16 SOMs utilised in our study were the same as in 2006. They include two generic and one regional pain scales (McGill Pain Questionnaire, Visual Analogue Scale and Shoulder Disability Index); three back pain disability questionnaires (Oswestry Low Back Disability Questionnaire, Quebec Back Pain Disability Questionnaire and Roland Morris Questionnaire); two generic functional disability scales (Barthel Index and Functional Status Index) and one generic health status questionnaire (Short-Form Health Survey); two arthritis scales (Arthritis Impact Measure and Western Ontario McMaster Osteoarthritis Index); four stroke disability scales (Stroke Rehabilitation Estimate of Movement, Modified Motor Assessment Scale, Rivermead Mobility Index Assessment Scale and Chedoke McMaster Stroke Assessment) and one cerebral palsy motor function scale (Gross Motor Function Measure) (Akinpelu & Eluchie 2006).

### Procedure

Participants were sent copies of the questionnaire and a covering letter explaining the purpose of the study.

Descriptive statistics, namely means, standard deviations, frequencies and percentages, were used to analyse the data as appropriate. Data obtained in this study were compared with the data of the 2006 study (Akinpelu & Eluchie 2006). A binary logistic regression was performed for the familiarity with and utilisation of SOMs for the 2016 data. Five factors (age, sex, education, duration of practice and work setting) were entered into the regression. Forward stepwise conditional analyses were performed for each SOM. The alpha level was set at *p* = 0.05.

### Ethical considerations

Ethical clearance was obtained from the joint Institution Review Board of the University of Ibadan and University College Hospital, Ibadan (ethical clearance number UI/EC/14/0280).

## Results

Three hundred and eighty-two questionnaires were sent out and 183 were returned (response rate: 48%). The physical characteristics of the participants in the two studies are fairly comparable (Table 1). There were more male physiotherapists than female physiotherapists in both studies. More physiotherapists possessed postgraduate qualifications in 2016 than in the 2006 study. More physiotherapists (43%) in 2016 had more than 10 years of clinical experience, whereas 53% had 0–5 years clinical experience in 2006. The majority of respondents worked in government teaching hospitals and general hospitals in both studies.

**TABLE 1:** Participants characteristics: 2016 (*n* = 183) versus 2006 (*n* = 236).

Characteristics	2016 % (*n*)	2006 % (*n*)
**Sex**		
Male	55.6 (100)	51.7 (122)
Female	44.4 (80)	48.3 (114)
**Qualification**		
Diploma	3.3 (6)	3.4 (8)
BSc/BMR	67.6 (123)	81.4 (192)
MSc	25.3 (46)	14.0 (33)
PhD	3.8 (7)	1.3 (3)
**Years of practice**		
0–5 years	39.8 (72)	53.4 (126)
6–10 years	17.1 (31)	30.1 (71)
> 10 years	43.1 (78)	16.5 (39)
**Work setting**		
Primary health care	1.7 (3)	1.3 (3)
Teaching hospital	50.8 (92)	41.5 (98)
General/state hospital	13.8 (25)	17.8 (42)
Private clinic/hospital	5.0 (9)	15.3 (36)
Specialist hospital	5.0 (9)	15.3 (36)
Home and school for the handicapped	7.7 (14)	1.7 (4)
Physiotherapy training institute (university)	4.4 (8)	5.1 (12)
Sports centre	0.6 (1)	1.3 (3)
Other	11.0 (20)	0.8 (2)

Between 50% and 96% of Nigerian physiotherapists were familiar with eight SOMs (Oswestry Low Back Pain Disability Questionnaire, Stroke Rehabilitation Estimation of Movement, Gross Motor Function Measure, Modified Motor Assessment Scale, Arthritis Impact Measurement, McGill Pain Questionnaire, Barthel Index and Visual Analogue Scale). These big ranges of percentages suggest that the physiotherapists have varied familiarity with each of the eight SOMs. The Visual Analogue Scale and Gross Motor Function Measure were the most utilised by Nigerian physiotherapists.

Table 2 shows the association of physical characteristics (age, sex, duration of practice, education and work setting) of participants and familiarity with SOMs. Only nine SOMs showed significant associations with the factors entered. Duration of practice, sex and age were the most important significant factors for familiarity with SOMs. Participants were more likely to be six and three times more familiar with the Oswestry Low Back Pain Disability Questionnaire if they had 6–10 years’ experience (OR = 5.64, CI = 1.52–20.88) and > 10 years’ experience (OR = 2.55, CI = 1.18–5.52), respectively, compared with 0–5 years’ experience. Participants were also five times more likely to be familiar with the Stroke Rehabilitation Estimation of Movement when they had 6–10 years’ experience (OR = 5.40, CI = 1.81–16.12), whereas > 10 years were twice as familiar compared with 0–5 years’ experience. Female physiotherapists were less familiar with the Stroke Rehabilitation Estimation of Movement (OR = 0.33, CI = 0.17–0.67) and the Gross Motor Function Measure (OR = 0.36, CI = 0.17–0.77).

**TABLE 2:** Association of physical characteristics of the participants and familiarity with standardised outcome measures (*n* = 183).

Variable	OR	SE (OR)	CI
**Oswestry Low Back Pain Disability Questionnaire Duration of practice**			
0–5 years (reference)	-	-	-
6–10 years	5.64**	0.67	1.52–20.88
> 10 years	2.55*	0.39	1.18–5.52
**Stroke Rehabilitation Estimation of Movement Sex**			
Male (reference)	-	-	-
Female	0.33**	0.36	0.17–0.67
**Duration of practice**			
0–5 years (reference)	-	-	-
6–10 years	5.40**	0.56	1.81–16.12
> 10 years	2.30*	0.38	1.13–5.03
**Gross Motor Function Measure Sex**			
Male (reference)	-	-	-
Female	0.36**	0.39	0.17–0.77
**Modified Motor Assessment Scale Sex**			
Male (reference)	-	-	-
Female	0.44*	0.37	0.22–0.90
**Duration of practice**			
0–5 years (reference)	-	-	-
6–10 years	4.97*	0.68	1.32–18.71
>10 years	1.45	0.38	0.69–3.08
**Western Ontario McMaster Osteoarthritis Index**			
Age	0.95*	0.03	0.90–0.99
**Rivermead Mobility Index assessment**			
Age	1.05*	0.02	1.00–1.10
**Functional Status Index Duration of practice**			
0–5 years (reference)	-	-	-
6–10 years	5.42***	0.51	1.99–14.71
> 10 years	2.87**	0.39	1.35–6.11
**Shoulder Pain Disability Index Duration of practice**			
0–5 years (reference)	-	-	-
6–10 years	3.12*	0.49	1.20–8.12
> 10 years	2.72**	0.37	1.32–5.61
**SF-36 Health Survey**			
Age	0.87**	0.05	0.80–0.95

OR, odd ratio; SE, standard error, CI, confidence interval.

* , significant at 0.05;

** , significant at 0.01;

*** , significant at 0.001.

Only age and sex were important significant factors for the utilisation of SOMs (Table 3). Participants were more likely to utilise the Oswestry Low Back Pain Disability Questionnaire, Roland Morris Questionnaire, Rivermead Mobility Index Assessment, Functional Status Index, Shoulder Pain Disability Index and Chedoke McMaster Stroke Assessment with a 1 year increase in age. Female physiotherapists were less likely to use the Stroke Rehabilitation Estimation of Movement, Gross Motor Function Measure and Modified Motor Assessment Scale.

**TABLE 3:** Association of physical characteristics of the participants and utilisation of standardised outcome measures (*n* = 183).

Variable	OR	SE(OR)	CI
**Oswestry Low Back Pain Disability Questionnaire**			
Age	1.05*	0.02	1.01–1.10
**Stroke Rehabilitation Estimation of Movement (Sex)**			
Male (reference)			
Female	0.30*	0.47	0.12–0.75
**Gross Motor Function Measure (Sex)**			
Male (reference)			
Female	0.46*	0.34	0.24–0.89
**Modified Motor Assessment Scale (Sex)**			
Male (reference)			
Female	0.49*	0.35	0.25–0.97
**Roland Morris Questionnaire**			
Age	1.07*	0.03	1.01–1.13
**Rivermead Mobility Index assessment**			
Age	1.07**	0.03	1.02–1.16
**Functional Status Index**			
Age	1.10***	0.03	1.05–1.16
**Shoulder Pain Disability Index**			
Age	1.10***	0.03	1.05–1.16

OR, odd ratio; SE, standard error; CI, confidence interval.

* , significant at 0.05;

** , significant at 0.01;

*** , significant at 0.001.

**FIGURE 1:**
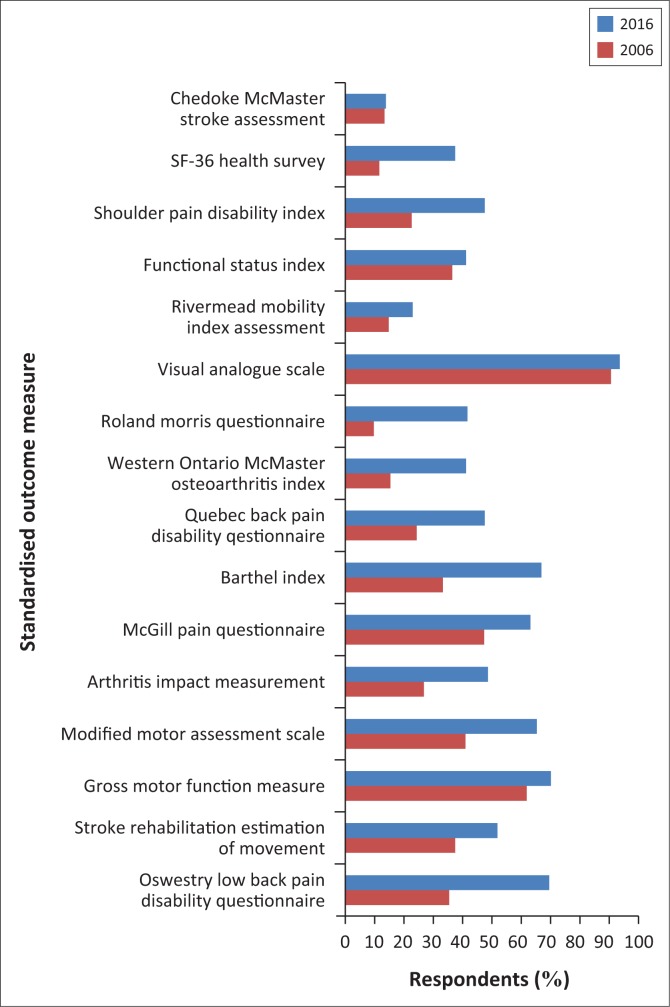
Familiarity with standardised outcome measures in Nigeria: 2016 (*N* = 183) versus 2006 (*N* = 236).

Figure 1 shows the comparison in familiarity with 16 SOMs between the 2016 and 2006 data. There was a difference in familiarity with all SOMs in 2016. Between 52% and 90% of physiotherapists were not familiar with 14 SOMs in 2006 (familiar with Visual Analogue Scale and Gross Motor Function Measure), whereas 51.4% – 85.8% of physiotherapists were not familiar with eight SOMs (Quebec Back Pain Disability Questionnaire, Shoulder Pain Disability Index, Western Ontario McMaster Osteoarthritis Index, Roland Morris Questionnaire, Rivermead Mobility Index Assessment, Functional Status Index, SF-36 Health Survey, Chedoke McMaster Stroke Assessment) in 2016.

**FIGURE 2:**
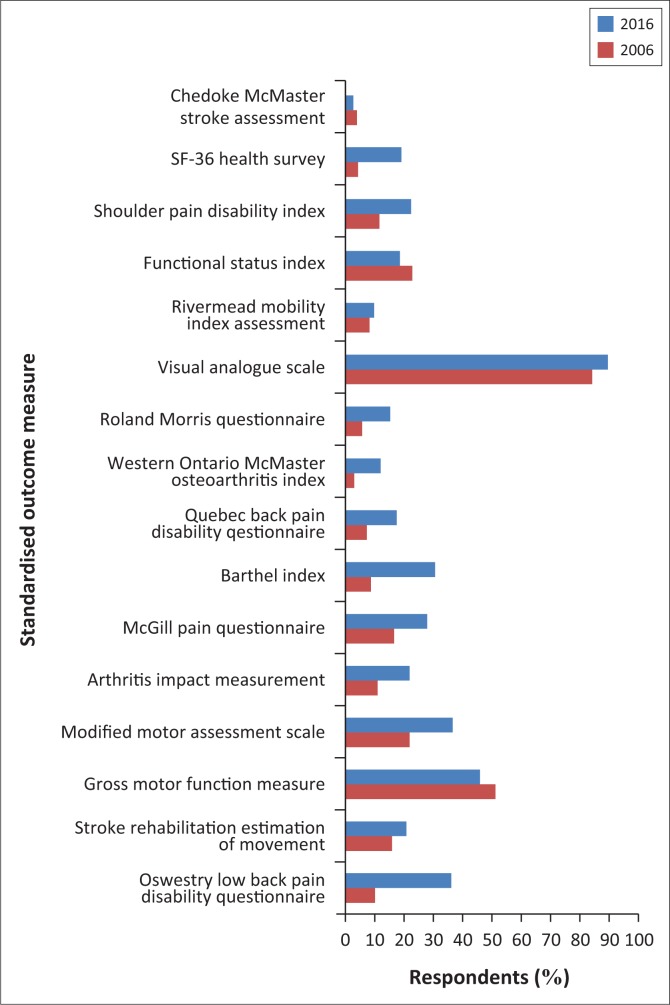
Utilisation of standardised outcome measures in Nigeria: 2016 (*N* = 183) versus 2006 (*N* = 236).

There was also an improvement in the utilisation of SOMs in 2016 (Figure 2) with the exception of the Chedoke McMaster Stroke Assessment, Functional Status Index and Gross Motor Function Measure. Apart from the Visual Analogue Scale and The Gross Motor Function Measure, 77% – 97% of physiotherapists were not utilising SOMs in the 2006 data, while in 2016, between 63.4% and 97.3% did not utilise SOMs. The least utilised SOMs in 2006 were the Western Ontario McMaster Osteoarthritis Index, Chedoke McMaster Stroke Assessment and SF-36 Health Survey, while in 2016, it was only the Chedoke McMaster Stroke Assessment. The Visual Analogue Scale and the Gross Motor Function Measure remained the most utilised in both the 2016 and 2006 data.

The respondents’ mean knowledge of administration of SOMs improved in 2016. It was 3.67 ± 2.51 in 2016 and 3.11 ± 2.52 in 2006.

## Discussion

This study sought to evaluate changes in physiotherapy practice in Nigeria on the utilisation of SOMs as well as physiotherapists’ familiarity with and knowledge of SOMs over the past decade to 2016. There was an improvement in their familiarity with and utilisation of 16 SOMs compared with the 2006 data. Differences in survey instruments and the listed SOMs make direct comparison difficult between the present study and existing published studies. However, the observation in this study was similar to the experience of Irish physiotherapists who had an improvement in their utilisation of outcome measures over a period of 5 years (Stokes & O’Neill 2008). The improvement in our study can be attributed to greater clinical experience and higher qualifications of physiotherapists compared with the 2006 data. It has been reported that clinicians with more clinical experience and qualifications tend to be more familiar and utilise SOMs (Copeland et al. 2008). Although there was an improvement in the utilisation of 13 SOMs in the data, 63.4% – 97.3% of physiotherapists were not utilising SOMs. This figure is worrisome and is an indication that Nigerian physiotherapists have not fully embraced the policy of the World Confederation for Physical Therapists on the integration of SOMs. Although the focus of this study was not on factors that hinder utilisation of SOMs, studies from other countries have suggested factors that might hinder the utilisation of outcome measures. These include cost, practicality, clinical relevance and a lack of knowledge over which outcome measures to choose and their use, lack of resources, lack of time, availability, lack of management support, lack of training and feasibility of measurement instruments (Duncan & Murray 2012; Meadows, Rogers & Greene 1998; Swinkels et al. 2011). Some of these factors can be looked into by the regulating body in Nigeria so that Nigerian physiotherapists can be encouraged to enhance their utilisation of outcome measures. Action can also be taken by professional organisations, teams and individuals if routine outcome measurement is to be achieved in Nigeria.

The knowledge of administration of SOMs also improved in our study compared with the 2006 data. Again, the increase in qualifications of Nigerian physiotherapists as suggested by the current data might be responsible for this. More physiotherapists now have Master’s and Doctor of Philosophy (PhD) qualifications. A link between having Master of Science and PhD degrees and knowledge of outcome measures has been suggested (Copeland et al. 2008). It has been postulated that postgraduate studies, dissertations and theses involve undertaking research, and if such research activities are experimental and clinical, it is most likely that outcome measures will be used in the studies. This may result in improved familiarity with outcome measures so that it becomes easier for physiotherapists to integrate them into routine clinical practice (Copeland et al. 2008). Although there was an improvement in the knowledge of administration of SOMs, the mean value of 3.67 out of 16 is unsatisfactory. Again, this low level of knowledge of outcome measures might be responsible for the high proportion of physiotherapists not utilising SOMs in our study. With the inclusion of courses on health measurement and outcome measures in the undergraduate physiotherapy curriculum in Nigeria, it is expected that the level of knowledge of SOMs would be better than the findings of this study suggest. A plausible reason for this finding may be that some of the listed 16 SOMs in this study might not be outcome measures taught in some of the training institutions in Nigeria. There is a need for a published compendium of SOMs which will serve as a guide for curriculum development of courses on outcome measures in physiotherapy training institutions in Nigeria. This will also ensure uniformity in the curricula of the various physiotherapy training institutions.

A longer duration of practice or clinical experience demonstrated the strongest association with the familiarity of outcome measures. This may be so, given that some of those who had more years of experience had postgraduate qualifications. Some of them might have used outcome measures in their research projects which might have resulted in more familiarity with SOMs. This is similar to a study that showed that advanced certification and knowledge of guidelines appeared to play a role in the clinical practice of physiotherapists treating patients with whiplash-associated disorders (Corkery, Edgar & Smith 2014).

Although there were improvement in knowledge of, familiarity with and utilisation of SOMs, it is still unsatisfactory. Although Akinpelu and Eluchie (2006) did not give a rationale for the selection of the 16 SOMs tested, a glance at the burden of disability listed by the Institute for Health Metrics and Evaluation in Nigeria (http://www.healthdata.org/nigeria) seems to support the expectation that practising physiotherapists in Nigeria should be aware of the 16 SOMs. Similar efforts as those made by other countries could be employed among Nigerian physiotherapists to improve their knowledge of and utilisation of SOMs. Strategies such as regular clinical auditing and local feedback have been shown to promote good clinical practice, adherence to guidelines and utilisation of SOMs (Bland et al. 2013). Also, developing toolkits and a tailored educational programme based on a thorough problem analysis have proved feasible and shown a positive effect on physiotherapists’ ability to choose one of many possible outcome measures in addition to their use of outcome measures in daily physiotherapy practice (Swinkels et al. 2015). Thus, the Association of Clinical and Academic Physiotherapists of Nigeria (ACAPN) and the NSP could invest in developing toolkits and tailored educational programmes to facilitate the implementation of clinical practice guidelines and utilisation of SOMs among their members. Such educational programmes should consist of lectures, educational seminars, peer support and online publications. Encouragement by ACAPN and NSP on the provision of advanced specialisation and professional development for physiotherapists could also go a long way in promoting utilisation of SOMs in Nigeria.

## Conclusion

The findings of this study suggest that there is an improvement in the familiarity with, knowledge of and utilisation of SOMs among Nigerian physiotherapists over the past decade of 2006–2016.
